# Repeated Δ-9-Tetrahydrocannabinol administration dose dependently increases stablished schedule-induced drinking

**DOI:** 10.1007/s00213-024-06563-3

**Published:** 2024-02-28

**Authors:** Esmeralda Fuentes-Verdugo, Ricardo Pellón, Miguel Miguéns

**Affiliations:** Departamento de Psicología Básica I, Facultad de Psicología, Universidad Nacional de Educación a Distancia (UNED), C/ Juan del Rosal 10, Ciudad Universitaria, Madrid, 28040 Spain

**Keywords:** THC, Cannabinoids, Schedule-induced behaviour, Time estimation, Habit

## Abstract

**Rationale:**

Schedule-induced drinking (SID) reproduces an excessive and repetitive behavioural pattern that has led to propose this procedure as an animal model to study compulsive behaviours. Although it is known that cannabis can cause several adverse effects, in recent years there has been great interest in the medical application of cannabis derivatives for obsessive-compulsive related disorders.

**Objectives:**

The present study investigated the effects of repeated THC administration on rates of previously acquired SID, as well as the possible alteration of its temporal distribution along inter-food intervals.

**Methods:**

Male Wistar rats acquired SID under a 30 min fixed-time 30-sec food delivery schedule (from 30 to 43 sessions to reach a stable level). Thereafter, 5 or 10 mg/kg daily i.p. injections of THC or vehicle were repeatedly administered for 7 days to evaluate the effects on SID.

**Results:**

Repeated THC administration at a dose of 5 mg/kg resulted in an increase on licking. Surprisingly, no effects on SID were observed with the 10 mg/kg dose. However, magazine entries were reduced with both THC doses. THC also modified the temporal distributions of licking and magazine entries during inter-food intervals.

**Conclusions:**

The present results show that repeated THC administration may (i) increase induced licking at moderate doses, (ii) reduce magazine entries, and (iii) affect the temporal pattern of SID. These findings suggest that THC does not appear to be beneficial to reduce compulsive behaviour in this animal model, while another collateral effect of THC —such as a greater habitual-like behaviour— needs to be considered.

## Introduction

Obsessive Compulsive and Related Disorders (OCRDs) involve several conditions identified by the behavioural manifestation of “compulsions”, i.e., repetitive and stereotyped acts without apparent adaptive function and performed in excess to reduce unpleasant consequences (Fineberg et al. 2018). Obsessive Compulsive Disorder (OCD) is the most recognizable mental health condition that manifests such compulsions, which are also observed in cases of Tourette’s disorder, substance addiction, problematic internet usage, trichotillomania, pathological gambling, schizophrenia or eating disorders (DSM-5; American Psychiatric Association 2013; Fineberg et al. 2018; Ioannidis et al. 2016; Swets et al. 2014). Compulsive behaviour can be shown in animal models of: (i) persistence to deal with adverse consequences; (ii) behavioural inflexibility; or, (iii) inability to stop behaviour (Maio et al. 2014). Compulsive symptoms are commonly treated pharmacologically with selective serotonin reuptake inhibitors (SSRI) and antipsychotics (Fineberg et al. 2010), and the fact that these drugs can reduce the compulsive behaviour mimicked by these models indicates predictive validity (Moreno and Flores 2012).

Schedule-induced drinking (SID) reproduces an excessive and repetitive behavioural pattern that has been recognized as the most robust and replicable animal model of compulsivity (Banasikowski and Hawken 2019; Moreno and Flores 2012). Excessive water intake occurs when food-deprived animals are exposed to schedules of intermittent food delivery, while having continuous access to water. The animals are not thirsty, and they do not have to drink to obtain the food. As well as SID, other behaviours have been included in the large category of adjunctive behaviours (Falk 1971; Roper 1981). SID has been documented in mice, rats, pigeons, monkeys, and humans, which demonstrates generality across species (Banasikowski and Hawken 2019; Pellón et al. 2020).

SID occurs as an inverted U-shaped distribution during inter-food intervals (Falk 1966). In this regard, it has been proposed that adjunctive behaviours allow organisms to discriminate time (Killeen and Fetterman 1988) and facilitate learning of time estimation tasks (Ruiz et al. 2016; Segal and Holloway 1963). These findings have generated theoretical interest in the temporal features of adjunctive behaviours to study the drug effects on the temporal distribution of SID (Flores and Pellón 1997; Pellon et al. 2007; Pellón and Blackman 1992; Íbias et al. 2016; 2017). However, the studies that consider SID as an animal model of compulsivity focus on testing the therapeutic potential of drugs typically used to treat the symptoms of OCRDs. The excessive drinking induced by this procedure is reduced by most of the substances used for the treatment of OCRDs symptoms, which indicates that the model shows predictive validity (Moreno and Flores 2012). This reduction occurs in the case of antipsychotics like haloperidol (Mittleman et al. 1994), pimozide (Snodgrass and Allen 1989), or clozapine (Didriksen et al. 1993), and some SSRIs (Hogg and Dalvi 2004; Woods et al. 1993), among others. However, in clinical practice, not all patients respond adequately to these treatments (Franklin and Foa 2011).

In recent years, despite their harmful effects (Volkow et al. 2016), cannabinoids have generated a great deal of interest because of their potential therapeutic use for psychiatric disorders, such as anxiety, sleep disorders, mood disorders, or post-traumatic stress disorder (see Sarris et al. 2020 for review). The psychoactive effects of cannabinoids are mediated principally by Δ9-tetrahydrocannabinol (THC) that acts as an agonist at the type-1 cannabinoid (CB1) receptor, a central component of the endocannabinoid system (ECS), primarily expressed in the central nervous system, but also expressed —together with the type-2 cannabinoid (CB2) receptor— in the peripheral nervous system and other peripheral organs and tissues (Iversen 2003; Matsuda et al. 1990; McCarberg and Barkin 2007; Munro et al. 1993). Elimination half-lives for THC are in the range of 20–30 h (Grotenhermen 2003), although it is also subjected to enterohepatic recirculation, which contributes to lengthen the duration of its effects for several days (Ashton 1999; Huestis 2007).

Recent research in both humans and animals has highlighted the role of the ECS in compulsive-like behaviours (Kayser et al. 2019). OCD has been associated with dysfunctions of the cortico-striato-thalamo-cortical circuit (Saxena and Rauch 2000) in structures that regulate goal-directed and repetitive/habitual behaviours (Burguiere et al. 2015; Langen et al. 2011; Lutz et al. 2015). CB1 receptors are present in regions of this circuitry, such as the prefrontal and anterior cingulate cortices, striatum, and substantia nigra (Díaz-Alonso et al. 2012; Goodman and Packard 2015; Harkany et al. 2007). Most preclinical studies investigating the effects of cannabinoids in OCD have employed the marble burying test, with receptor CB1 agonists such as WIN 55,212-2, anandamide or cannabidiol decreasing the number of buried marbles (Deiana et al. 2011; Umathe et al. 2012). However, no relevant effects on SID were found using the cannabinoids cannabidiol or WIN 55,212-2 as potential novel treatments (Martín-González et al. 2018). By contrast, recent research in our laboratory found that chronic THC administration delayed SID acquisition, and acute THC administration reduced SID only in rats that had previously experienced a chronic treatment with the same drug, suggesting a sensitization-like effect (Fuentes-Verdugo et al. 2022). Clinical trials have shown that cannabis users with OCD reported that cannabinoids mitigated their symptoms, including obsessions and compulsions (Müller-Vahl 2013; Schindler et al. 2008; Szejko et al. 2020; Patel et al. 2021), and another recent study reported that nabilone, a synthetic cannabinoid with similar effects to THC, alleviated OCD severity in combination with exposure and response prevention therapy, nearly doubling the effect compared to exposure and response prevention alone (Kayser et al. 2020). All this evidence points to ECS as a potential target for novel medications aiming OCD symptoms.

In the present study, we tested the potential therapeutic role of THC in the compulsive drinking that is commonly developed in the SID procedure. We investigated the repeated administration of two different THC doses (5 and 10 mg/kg) for 7 days in succession, a treatment that has been shown to induce tolerance and to reduce the density of cannabinoid receptors in the striatum and limbic forebrain (Rodríguez de Fonseca et al. 1994). The treatment was applied about 1 h after the behavioural test, and not before it, considering its long-lasting duration. The results will be discussed in terms of the implications of the use of THC as a treatment for compulsive psychiatric disorders.

## Materials and methods

### Subjects

Experimentally naïve male Wistar rats (*n* = 32; age, 7 weeks) from Charles River laboratories (Lyon, France) were used in this study. They were firstly housed four in a cage for a week to get acquainted with the animal facilities. The room environment was maintained on a 12-hour light/dark cycle (from 8:00 am to 8:00 pm), in a controlled ambient temperature (20 ± 2 ºC), with approximately 55% relative humidity. The animals initially had free access to both food and water. They were later (age, 8 weeks; weight, 257 ± 15 g) singly housed in clear polycarbonate cages (18 × 32 × 20.5 cm) and gradually food restricted (see Section “*SID acquisition*”). The rats were treated in accordance with European Union Council Directive 2010/6, Spanish Royal Decree 53/2013 for the use of animals in research, the corresponding authorization from the Community of Madrid (PROEX 077/18) and UNED bioethics committee.

### Apparatus

The experiment was conducted in eight conditioning chambers (Letica LI-836; Letica Instruments customized by Cibertec, Madrid, Spain). The chambers (29 × 24.5 × 35.5 cm) were housed inside sound-attenuating wooden cabinets that contained a ventilation system and a small observation window at the front. The chamber walls were made of polycarbonate, except for the front wall made of modular aluminium panels. The floor consisted of metal rods. In the right wall, 7 cm above the floor, there was an aperture of 3.2 × 3.9 cm to place a bottle of water. Licks at the spout of the bottle closed an electric circuit with the metal rods of the chamber floor, which allowed the licking to be recorded. An automatic food dispenser delivered 45 mg food pellets (Bio-Serv, Flemington, NJ, USA) into a food tray placed 3.7 cm from the floor, between the two retracted levers of the panel. A photo beam sensor was positioned across the food tray to measure head entries. The chambers had two inactive 3 W lamps above each lever, and an indirect 25 W houselight inside the wooden cabinet that was lit during the sessions. The sound of 60 dB produced by the ventilation system masked any exterior noise. Spout licks and magazine entries were recorded by a PC computer running MED-PC-IV (MED Associates, Inc., Fairfax, VT, USA).

### SID acquisition

The rats’ weights were gradually reduced by moderate food restriction until they stabilized at 85% (age, 10 weeks) of their free-feeding weights with reference to a standard growth curve for the Wistar strain. The animals were weighed daily before the experimental sessions and fed at least 20 min after their completion to maintain the weight criterion. The procedure consisted of a fixed time (FT) 30-second schedule in which a food pellet was regularly dispensed every 30 s for a 30-minute duration each session. All rats received the same amount of food in each session (60 food pellets) and water was freely available. The bottles were filled with fresh tap water and placed in the chambers before each experimental session. This training lasted between 30 and 43 sessions until all animals reached stable levels of performance (stability criterion: 5 consecutive days with a variation with respect to the mean not exceeding ± 25%).

After SID acquisition, the average number of licks carried out by each animal was calculated based on the last five SID sessions of each individual rat. Using these averages, the subjects were matched based on their licking rates and then within each triad randomly assigned to the experimental conditions to ensure comparable means among groups —Vehicle (*n* = 10), 5 mg/kg THC (*n* = 11), and 10 mg/kg THC (*n* = 11).

The water consumption in the home cage was also measured to control for prandial intake.

### Drug preparation

THC (THC Pharm Gmbh; Main/Frankfurt, Germany) was prepared daily in aliquots for final concentrations of 5 mg/ml and 10 mg/ml, in a vehicle of absolute ethanol (Emsure Merck KGaA; Darmstadt, Germany), cremophor (KolliphorEL; Sigma Aldrich Co.; St. Louis, MO, USA), and saline (0.9% sodium chloride) in a ratio of 1:1:18. The ethanol concentration in the THC and vehicle solutions was 5%, with ethanol doses of 0.02 g/kg. The drug was preserved with N_2_, protected from light, and frozen (-35 ºC) until administration.

### Repeated THC administration tested on SID

The pharmacological procedure was conducted after all the animals had achieved stability in SID, initiating the treatment phase on the same day. The first control session consisted of the injection of a saline solution to all animals to rule out any unexpected effect of intraperitoneal injections (i.p.). During the following 7 sessions, the animals were treated according to the group condition assigned: Vehicle (0 mg/kg THC), 5 mg/kg THC, or 10 mg/kg THC. Injections were intraperitoneally administered in a volume of 1 ml/kg body weight 1 h after the end of SID sessions to avoid acute THC effects on behaviour. The behavioural SID procedure was as in the acquisition phase (see Section “2.3. *SID acquisition*” above). Over the 7 sessions of drug administration, water consumption in the home cage was also measured to compare it with the last seven sessions taken before treatment to rule out a general effect of THC on the animals’ thirst.

### Statistical analysis

**Fig. 1:**
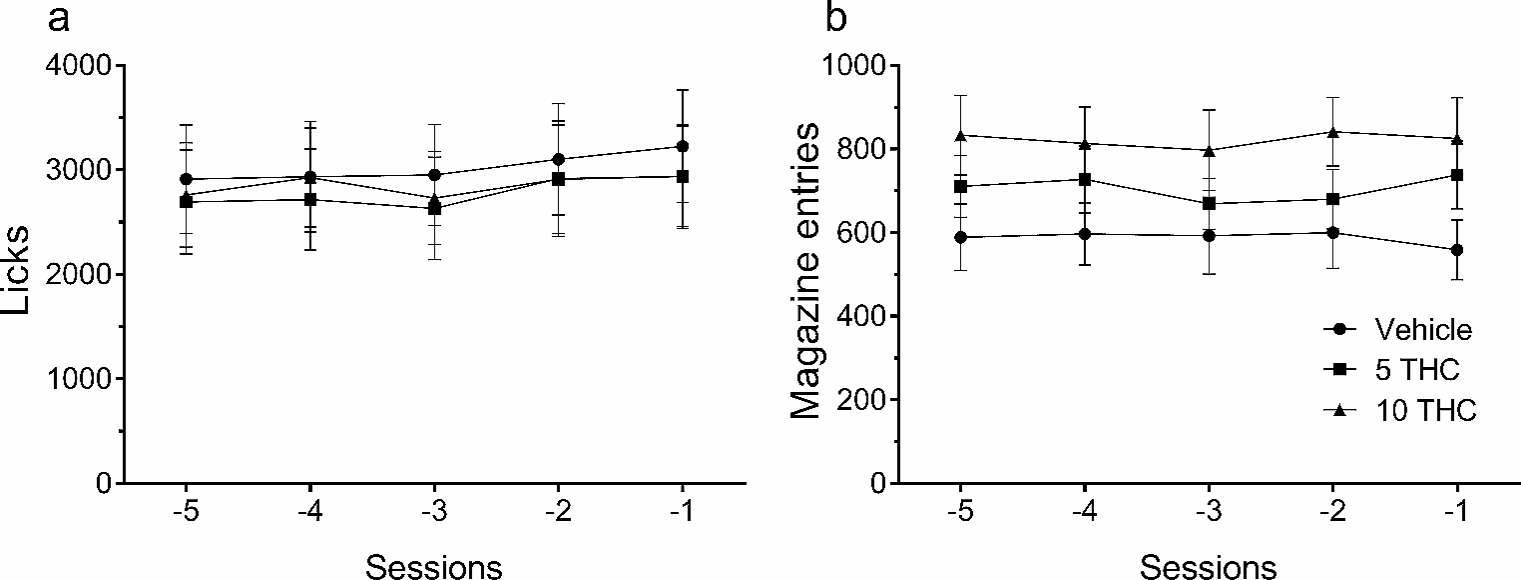
Licks and magazine entries after SID acquisition. Total number of licks (**a**) and magazine entries (**b**) for vehicle (circles, *n* = 9) or THC treated animals, at a dose of 5 mg/kg (squares, *n* = 10) or 10 mg/kg (triangles, *n* = 10), during the last five acquisition sessions, established as the baseline to transform the data (mean ± SEM).

Licks and magazine entries were previously transformed into a percentage for each subject with respect to the average of the last five stable acquisition sessions of SID (Fig. 1), and the mean was used to obtain the percentage of change.

Data were analysed using a mixed-design analysis of variance (ANOVA), with a between-subjects factor ‘treatment’ (vehicle, 5 mg/kg THC, and 10 mg/kg THC), and a within-subjects repeated measures factor ‘session’ with one level for each experimental session. Bonferroni correction was conducted in post-hoc comparisons with statistically significant *p* values of α < 0.05. Effect sizes were calculated using partial eta squared (*η*_*p*_^*2*^). Sphericity principle violations were corrected using Greenhouse-Geisser (GG) epsilon (*ε*) to adjust the degrees of freedom (α < 0.05) with the Mauchly Sphericity test. Outliers were identified using the interquartile range criterion, and the final groups were: Vehicle (*n* = 9), 5 mg/kg THC (*n* = 10), and 10 mg/kg THC (*n* = 10). All analyses were conducted using SPSS 24 © software.

SID temporal distribution was studied using the trapezoidal rule for the area under the curve. The parameters obtained with this analysis are the highest point of the x-axis (representing the peak time), the highest point of the y-axis (representing the peak percentage of licks or entries), and the total area of the temporal distribution as a function of time and percentage of licks or entries.

## Results

The mean total licks (± SEM) of each group over the last 5 sessions of SID taken after constituting the groups were 3025.1 ± 520.5, 2779 ± 528.9, and 2853.3 ± 508.1, for vehicle, 5 mg/kg THC, and 10 mg/kg THC groups, respectively. During these sessions, when SID reached stability, no statistically significant differences in mean total licks were found (*F*_4,8_= 2.33, *p* = .143, *ηp²*= 0.31). The control measure taken to evaluate the effect of THC treatment on regular thirst did not show statistical differences with respect to the preceding measure taken without drug administration for 7 days (*F*_2,26_ = 1.35, *p* < .256, *η*_*p*_^*2*^ = 0.05; descriptive data, mean ± SEM in Table 1).

**Table 1:** THC effects on total water intake in the home cages (23 h 30 min) over 7 days prior to treatment and 7 days of treatment with vehicle (*n* = 9), 5 mg/kg THC (*n* = 10) or 10 mg/kg THC (*n* = 10)

	Water intake (ml)		
	Vehicle	THC (5 mg/kg)	THC (10 mg/kg)
Prior to treatment	22 ± 2.5	22.48 ± 2.3	21.7 ± 2.7
During treatment	23.9 ± 2.5	22 ± 2.19	21.9 ± 2.27

Data expressed as the mean ± SEM

**Fig. 2:**
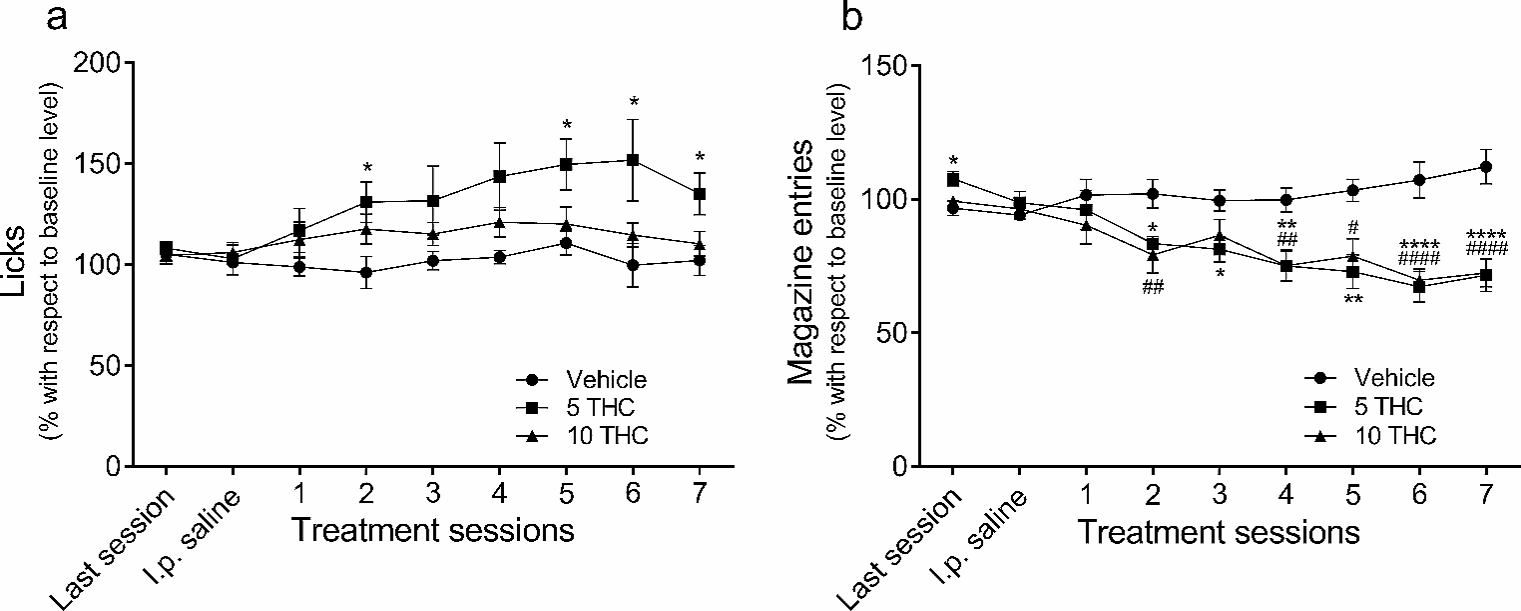
Effects of repeated administration of THC on licks and magazine entries. The effects on SID are shown for repeated administration of vehicle (circles, *n* = 9) or THC, at a dose of 5 mg/kg (squares, *n* = 10) or 10 mg/kg dose (triangles, *n* = 10). Data show the percentage of licks (**a**) and magazine entries (**b**) with respect to baseline level. The data represent the mean ± SEM of the last acquisition session, the control session with a previous saline i.p. injection, and the 7 consecutive sessions with a treatment injection administered 1 h after the test. Vehicle vs. 5 THC: * *p* < .05, ** *p* < .01, **** *p* < .0001. Vehicle vs. 10 THC: # *p* < .05, ## *p* < .01, #### *p* < .0001; using Bonferroni post-test

The effects of THC on licks and magazine entries are shown in Fig. 2. The data (mean ± SEM) are expressed as percentage of change with respect to the last 5 SID training sessions. This figure includes data for the last SID acquisition session, the session in which all animals were i.p. injected with saline solution, and the 7 consecutive sessions where all animals were i.p. injected with a single injection of vehicle, 5 mg/kg THC or 10 mg/kg THC. THC treatment significantly affected licks (*treatment*, *F*_2,26_ = 3.45, *p* < .047, *η*_*p*_^*2*^ = 0.21; *treatment x session* interaction, *F*_7,95_ = 2.43, *GG* (*ε*) = 0.46, *p* < .023, *η*_*p*_^*2*^ = 0.16; *session*, *F*_4,95_ = 5.32, *p* < .001, *η*_*p*_^*2*^ = 0.17). Bonferroni post-hoc analysis revealed that THC only increased licking in the rats treated with the 5 mg/kg dose. This effect was more pronounced over the course of the sessions (Fig. 2a). There was also a significant effect on magazine entries in both groups of rats treated with THC (*treatment*, *F*_2,26_ = 10.12, *p* < .001, *η*_*p*_^*2*^ = 0.44; *treatment x session interaction*, *F*_7,95_ = 5.76, GG (*ε*) = 0.46, *p* < .0001, *η*_*p*_^*2*^ = 0.31; *session*, *F*_4,95_ = 5.32, *p* < .0001, *η*_*p*_^*2*^ = 0.17). Post-hoc analysis revealed that the more days of THC treatment there were, the greater the reduction of magazine entries percentage in both groups treated with the drug (Fig. 2b).

**Fig. 3:**
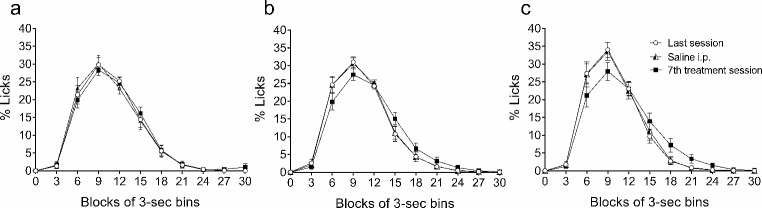
Temporal distribution of SID. Percentage of licks with respect to the total number of licks performed in the inter-food interval for each rat throughout successive 3-sec bins in animals pre-treated with vehicle (**a**) or THC, at a dose of 5 mg/kg (**b**) or 10 mg/kg (**c**). Data represent the mean ± SEM of the last session (circles), the control successive session with a previous saline i.p. injection (triangles), and the 7th session of treatment (squares)

**Fig. 4:**
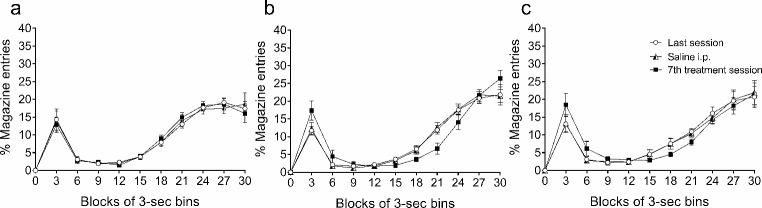
Temporal distribution of magazine entries. Percentage of magazine entries with respect to the total number of entries performed in the inter-food interval for each rat throughout successive 3-sec bins in animals pre-treated with vehicle (**a**) or THC, at a dose of 5 mg/kg (**b**) or 10 mg/kg (**c**). Data represent the mean ± SEM of the last session (circles), the control successive session with a previous saline i.p. injection (triangles), and the 7th session of treatment (squares)

The effects of the repeated administration of the vehicle solution and the 5 and 10 mg/kg doses of THC on the temporal distribution of licks (Fig. 3) and magazine entries (Fig. 4) are represented in successive 3-sec bins during the 30 s of the inter-food interval in the form of percentage (mean ± SEM) of the total number of licks or entries made during the inter-food interval for each rat. The descriptive parameters obtained with the area under the curve were the peak time, the peak percentage of licks or entries, and the total area of the temporal distribution, as a function of time and percentage of licks or magazine entries (data in Tables 2 and 3 for licks and magazine entries, respectively).

The temporal distribution of licks showed the common SID pattern with maximal responding at 9 s of the inter-food interval in all cases. The treatment did not affect the peak time. The peak percentage of licks was not affected by the saline injection, but THC slightly decreased this parameter (5% at the 5 mg/kg dose, and 6% at the 10 mg/kg dose), although licking was maintained higher and longer from second 15 to 21 (see Fig. 3). The total area of the temporal distribution as a function of time and percentage of licks was not affected in any group (see Table 2).

**Table 2:** Descriptive parameters obtained with the area under the curve of the temporal distribution of licks

Sessions	Peak time (sec)			Peak licks (%)			Total area (% ± SEM)		
	Vehicle	THC (5 mg/kg)	THC (10 mg/kg)	Vehicle	THC (5 mg/kg)	THC (10 mg/kg)	Vehicle	THC (5 mg/kg)	THC (10 mg/kg)
Last	9	9	9	30	31	34	300 ± 30 29.4	300 ± 27 2746.6	300 ± 31
Saline	9	9	9	30	31	34	300 ± 34	300 ± 26 339.3	300 ± 32
Treat. (7th day)	9	9	9	30	26 (-5)	28 (-6)	299 ± 26	299 ± 29 39.7	300 ± 43

Percentage difference on 7th day of treatment respect to the other sessions of reference is indicated in brackets

The temporal distribution of entries displays a shape that is almost the opposite to that of the SID distribution. Maximal responding occurred in proximity to food delivery, both between bins 27 and 30 before food delivery, and 3 s after its occurrence. By contrast, the minimum percentage of entries occurred when the peak of licks peaked (see Fig. 4). The peak of entries across sessions was similar for the groups treated with vehicle or 10 mg/kg THC; however, for the group treated with 5 mg/kg THC, the peak was 5% higher on the 7th day of treatment in comparison to the other sessions. No differences were found between the last session and the saline control session for any group for this or the other parameters. The total area of the temporal distribution as a function of time and percentage of entries was only slightly lower for the group treated with 5 mg/kg THC; no differences for the other two groups were observed (see Table 3). In line with the extended licking after peaking caused by THC treatment, magazine entries remained low between bins 15 and 24.

**Table 3:** Descriptive parameters obtained with the area under the curve of the temporal distribution of magazine entries

Sessions	Peak time (sec)			Peak entries (%)					Total area (% ± SEM)		
	Vehicle	THC (5 mg/kg)	THC (10 mg/kg)	Vehicle	THC (5 mg/kg)		THC (10 mg/kg)		Vehicle	THC (5 mg/kg)	THC (10 mg/kg)
Last	27	30	30	19.13	21.84		20.68		276 ± 26 29.4	267 ± 24 29.4	269 ± 33
Saline	30	27	30	18.64	21.76		21.9		272 ± 31	268 ± 23	267 ± 34
Treat. (7th day)	27	30	30	18.65	26.37		21.41		276 ± 26	260 ± 33	268 ± 36

Mean ± SEM

## Discussion

This study aimed to investigate the effects of repeated THC administration on SID, a putative animal model of compulsivity, and showed that THC increased licking when animals were successively treated with a dose of 5 mg/kg —but not with the higher dose of 10 mg/kg, which had no significant effect. Moreover, the treatment with both doses resulted in a general decrease on magazine entries —an effect which became more pronounced as the sessions progressed.

The result that the 5 mg/kg dose of THC caused a significant increase on licking contrasts with previous findings that demonstrated the potential use of cannabinoids to reduce compulsive behaviour, as seen with the marble burying test in rodents after administration of different cannabinoid agonists such as WIN 55,212-2 or cannabidiol (Gomes et al. 2011; Nardo et al. 2013). Similar results were obtained on grooming behaviour induced by 2,5-dimethoxy‐4‐iodoamphetamine —an animal model of Tourette syndrome—, in which 5 mg/kg of THC inhibited grooming in juvenile and young adult mice (Gorberg et al. 2020). We have recently shown that THC delayed SID acquisition when it had been previously chronically administered, and reduced SID after being re-administered acutely (Fuentes-Verdugo et al. 2022). Thus, chronic THC administration delays SID acquisition but increased it when SID is steadily established before its administration. Moreover, the reduction of SID after acute administration seems to be due to the animals having previously experienced short-lived effects of THC. It is interesting to note that cannabinoids regularly result in “inverted-U” dose-response curves on the parameters tested in the elevated plus maze (Campos and Guimarães 2009; Moreira et al. 2007), as well as dose-dependent effects on spatial and object working memory (Verrico et al. 2012), dopamine release, anxiety, stress, conditioned taste aversion, and catalepsy (reviewed in Kubilius et al. 2018). In line with this, high doses of anandamide, a substance with similar properties to THC, increased the number of buried marbles compared with lower doses that reduced this behaviour, inducing both anti-compulsive and pro-compulsive effects via the transient receptor potential action channel V1 (TRPV1) receptors (Umathe et al. 2011, 2012). However, no significant effects on SID were observed in response to the acute administration of the cannabinoids AM404 or WIN55212-2, and only cannabidiol at the dose of 1 mg/kg resulted in a slight reduction of SID (Martín-González et al. 2018). Considering the pharmacological dose-response features of cannabinoids, this can serve as a basis to understand the different results that we obtained with the THC doses of 5 and 10 mg/kg. Given the slow-elimination profile of cannabinoids, THC effects may differ depending on the moment the behaviour is evaluated. It is possible that after one hour of a single acute administration, the 5 mg/kg dose of THC may reduce licking behaviour (Fuentes-Verdugo et al. 2022), but the residual effects (23 h after) of repeated administration of the same dose may produce opposing effects by increasing SID.

The varying effects of THC on SID acquisition and performance can be attributed to the intricate interactions between THC and the endocannabinoid system over different time frames and dosage accumulation. Within the brain, THC´s impact on presynaptic CB1 receptors primarily results in inhibitory effects on synaptic transmission (Lupica and Hoffman 2018). Signalling through these receptors contributes to its behavioural THC effects (reviewed in Augustin and Lovinger 2022). Consequently, these short-term actions may be responsible for the decline in SID shortly after acute THC administration. Nonetheless, chronic THC administration may trigger more complex neurobiological adaptations that could be related to delayed SID acquisition. The most consistent neurobiological consequence of chronic THC exposure is decreased CB1 receptor numbers, as well as diminished CB1 agonist and endocannabinoid-induced presynaptic modulation (reviewed in Hoffman et al. 2021; Kesner and Lovinger 2020). THC alters synaptic plasticity in several neuronal circuits that could be related to impaired cognition and behaviour (Hoffman et al. 2021). As mentioned in Fuentes-Verdugo et al. (2022), this disruption could impact the learning of the task. Moreover, once SID is stablished, prolonged exposure to THC may induce additional neuroadaptive changes, potentially resulting in an increase in SID as the system adjusts to the chronic presence of the substance. It is noteworthy that delayed acquisition observed in our previous study occurs after a clearance period when the animals were not exposed to the drug.

SID displays a distinctive temporal pattern in the form of an inverted U-shaped function as the time between food deliveries elapses (Pellón et al. 2020). During the last SID session in the present study, the temporal distribution of licking showed an identical shape when the saline injection session was compared with the last day of SID acquisition in any of the three groups of the experiment; vehicle administration did not affect it either. By contrast, the temporal distributions of licks in animals repeatedly treated with THC showed a slight reduction in their peaks of 5% and 6% with the 5 and 10 mg/kg doses, respectively (see Fig. 3b and c). However, the other parameters analysed (peak time and the total area under the curve) were not affected by THC (see Table 2). Both groups treated with THC reached lower peak licks kept licking longer, through the FT interval until second 21. A similar result was obtained when animals were chronically treated for 14 days with THC and tested on SID in a later phase (Fuentes-Verdugo et al. 2022). The peak percentage of licks was affected in the same way, and the animals kept licking longer instead of only drinking in the first few seconds immediately after the food delivery. The peak time was also delayed in Fuentes-Verdugo et al. (2022), so it seems that time estimation might be more affected after long, repeated treatments. The SID procedure has demonstrated its usefulness as a tool for assessing temporal estimation (Ruiz et al. 2016), hence the interest in testing the effects of different drugs on these temporal features. Cannabinoid users miscalculate the duration of time intervals and overestimate the duration of time (Lieving et al. 2006; McDonald et al. 2003; Sewell et al. 2012) — a result that agrees with the fact that the animals of the present study kept licking longer with THC than with vehicle. By contrast, rats exhibited a shortened response time and decreased sensitivity to time under the acute effects of the cannabinoid agonists THC or WIN 55,212–2 (Crystal et al. 2003; Han and Robinson 2001). Therefore, this shows that THC may affect time estimation, but these effects seem to be dependent on the nature of the task or the type of drug administration, i.e., acute or repeated.

The persistent licking during the inter-food interval seen in the animals treated with THC may reflect some features of habit-like behaviours. In this regard, cannabinoids influence the transition from volitional behaviour to habit formation, and chronic exposure to them induces structural plasticity in the dorsal striatum, a region closely involved in this kind of behaviour (Fernandez-Cabrera et al. 2018; Goodman and Packard 2015; Lovinger et al. 2010). Recent research has demonstrated that rats subjected to the SID model exhibited greater habitual-like behaviour (Merchán et al. 2019). Moreover, SID is also associated with increased dendritic spine density in dorsolateral striatum neurons (Íbias et al. 2015). All these findings lead us to suggest that THC administration possibly facilitates habit-like behaviours during the inter-food intervals on the SID procedure. Repeated administration of THC when SID has been acquired may therefore result in an increase of habitual performance, as was the case in the present study, while administering THC before the behaviour is acquired would not be expected to have the same effect; indeed, it resulted in a retardation of SID acquisition (Fuentes-Verdugo et al. 2022).

Magazine entries were also affected by THC administration, with a general decrease throughout sessions at both doses tested compared with animals that received vehicle (Fig. 2b). This seems to be caused by the persistence of licking throughout the FT interval in THC treated animals (see Figs. 3 and 4). Moreover, animals treated with THC had higher percentages of entries in bins 30 and 3 —the moments that were closer to the reinforcer delivery— with respect to the same bins in control sessions. This result is consistent with the explanation that all the behaviours generated within inter-food intervals compete for their manifestation during the interval and tend to organize in a sequential manner (Pellón et al. 2020). SID behaviour competes with magazine entries, lever presses, or wheel running, thus displaying different temporal patterns to one another (Gutiérrez-Ferre 2019; Pellón and Killeen 2015). Therefore, the persistence of THC treated rats on SID seems to decelerate the emergence of magazine entries.

Finally, it is important to note that in alignment with a previous study’s approach (Fuentes-Verdugo et al. 2022), our current experiments focused exclusively on male subjects. However, it is essential to recognize the differential effects of cannabinoids between males and females. Growing evidence suggests that pharmacological and hormonal differences may contribute to variations in responses to cannabinoid substances (see Cooper and Craft 2018 for review). Hence, future studies should include both male and female subjects to improve our understanding of the gender-specific effects of cannabinoids on SID.

## Conclusions

In conclusion, repeated THC administration affects putative compulsive licking on SID in a dose-dependent manner. Increases on licking were only evident with the 5 mg/kg dose. Moreover, THC effects on SID behavioural persistence modified the occurrence of magazine entries. THC is not postulated as a beneficial treatment for the compulsive symptoms that might manifest from SID given the results that we obtained with this model. Finally, it is important to note that the treatment applied could cause the opposite of what was sought, by increasing the persistence of the compulsive behaviour. More studies are warranted to study cannabinoids with this animal model to further identify their specific dose-dependent effects.
